# Supplementary material for: Antibiotic-Resistant Bacteria Isolated from Street Foods: A Systematic Review
Source: Antibiotics (Basel). 2024 May 23;13(6):481. doi: 10.3390/antibiotics13060481 (PMC11201236; doi:10.3390/antibiotics13060481)
Supplement: Supplementary file 1 [file antibiotics-13-00481-s001.zip › Supplementary File 2.pdf]

Supplementary file 2. Quality assessment of included studies

Reference	Quality Assessment Criteria Probing Questions (Q)									Study level quality score		Quality
	Q1	Q2	Q3	Q4	Q5	Q6	Q7	Q8	Q9	Total No Yes (Y)	Percentage of Yes (Y) %	
Adeleke & Owoseni, 2022 [43]	Y	Y	U	U	U	Y	Y	Y	Y	6	66.6	Moderate
Adhikari *et al.*, 2023 [44]	Y	Y	Y	Y	Y	Y	Y	Y	Y	9	100	High
Agyirifo *et al.*, 2023 [45]	Y	Y	Y	Y	Y	Y	Y	Y	Y	9	100	High
Beshiru & Igbinosa, 2023 [46]	Y	Y	Y	Y	Y	Y	Y	Y	Y	9	100	High
Campos *et al.*, 2015 [47]	Y	Y	U	Y	U	Y	Y	Y	Y	7	77.7	High
Chaje¸cka-Wierzchowska *et al.*, 2015 [48]	Y	Y	Y	Y	Y	Y	Y	U	U	7	77.7	High
Dela *et al.*, 2023 [49]	Y	Y	Y	Y	Y	Y	Y	Y	Y	9	100	High

Reference	Quality Assessment Criteria Probing Questions (Q)									Study level quality score		Quality
	Q1	Q2	Q3	Q4	Q5	Q6	Q7	Q8	Q9	Total No Yes (Y)	Percentage of Yes (Y) %	
Giri *et al.*, 2021 [50]	Y	Y	Y	Y	Y	Y	Y	Y	Y	9	100	High
Gurrurajan *et al.*, 2018 [51]	Y	Y	U	Y	Y	Y	Y	U	Y	7	77.7	High
Hasan *et al.*, 2021 [52]	Y	Y	U	Y	Y	Y	Y	U	Y	7	77.7	High
Johura *et al.*, 2020 [53]	Y	Y	U	Y	Y	Y	Y	U	Y	7	77.7	High
Lin *et al.*, 2017 [54]	Y	Y	Y	Y	Y	Y	Y	Y	Y	9	100	High
Mesbah, Mashak & Abdolmaleki, 2021 [55]	Y	Y	Y	Y	Y	Y	Y	Y	Y	9	100	High
Nikiema *et al.*, 2021 [56]	Y	Y	Y	Y	Y	Y	Y	Y	Y	9	100	High
Sivakumar *et al.*, 2019 [57]	Y	Y	Y	Y	Y	Y	Y	Y	Y	9	100	High
Yang *et al.*, 2017 [58]	Y	Y	Y	Y	Y	Y	Y	Y	Y	9	100	High

Reference	Quality Assessment Criteria Probing Questions (Q)									Study level quality score		Quality
	Q1	Q2	Q3	Q4	Q5	Q6	Q7	Q8	Q9	Total No Yes (Y)	Percentage of Yes (Y) %	
Zurita *et al.*, 2020 [59]	Y	Y	Y	Y	U	Y	Y	U	U	6	66.6	Moderate

Q1: Was the sample frame appropriate to address the target population?

Q2: Were study participants sampled in an appropriate way?

Q3: Was the sample size adequate?

Q4: Were the study subjects and the setting described in detail?

Q5: Was the data analysis conducted with sufficient coverage of the identified sample?

Q6: Were valid methods used for the identification of the condition?

Q7: Was the condition measured in a standard, reliable way for all participants?

Q8: Was there appropriate statistical analysis?

Q9: Was the response rate adequate, and if not, was the low response rate managed appropriately?
